# Extensive dataset of boar seminal plasma proteome displaying putative reproductive functions of identified proteins

**DOI:** 10.1016/j.dib.2016.07.037

**Published:** 2016-07-29

**Authors:** Cristina Perez-Patiño, Isabel Barranco, Inmaculada Parrilla, Emilio A. Martinez, Heriberto Rodriguez-Martinez, Jordi Roca

**Affiliations:** a Department of Medicine and Animal Surgery, Faculty of Veterinary Science, University of Murcia, Spain; b Department of Clinical & Experimental Medicine (IKE), Linköping University, Sweden

**Keywords:** Porcine, Seminal plasma, Proteome, Reproductive functionality

## Abstract

A complete proteomic profile of seminal plasma (SP) remains challenging, particularly in porcine. The data reports on the analysis of boar SP-proteins by using a combination of SEC, 1-D SDS PAGE and NanoLC-ESI-MS/MS from 33 pooled SP-samples (11 boars, 3 ejaculates/boar). A complete dataset of the 536 SP-proteins identified and validated with confidence ≥95% (Unused Score >1.3) and a false discovery rate (FDR) ≤1%, is provided. In addition, the relative abundance of 432 of them is also shown. Gene ontology annotation of the complete SP-proteome complemented by an extensive description of the putative reproductive role of SP-proteins, providing a valuable source for a better understanding of SP role in the reproductive success. This data article refers to the article entitled “Characterization of the porcine seminal plasma proteome comparing ejaculate portions” (Perez-Patiño et al., 2016) [1].

**Specifications Table**

**Table:** 

Subject area	*Reproductive biology*
More specific subject area	*Proteomics of boar seminal plasma*
Type of data	*Excel file*
How data was acquired	*Samples were analyzed using a NanoLC Ultra 1-D plus Eksigent (Eksigent Technologies, Dublin, CA, USA) directly connected to an AB SCIEX TripleTOF 5600 mass spectrometer (AB SCIEX, Framingham, MA, USA)*
Data format	*Processed, analyzed*
Experimental factors	*Boar seminal plasma from twice centrifuged ejaculates in order to remove sperm and obtain cell-free seminal plasma*
Experimental features	*Boar seminal plasma proteome description and relative quantification. The sample proteins were fractionated using SEC and 1-D SDS PAGE followed by trypsin digest to analyze less abundant proteins and in-solution digestion to analyze more abundant proteins*
Data source location	*Murcia (Spain)*
Data accessibility	*Data are available within this article and via the PRIDE partner repository with the dataset identifier* PRIDE: PXD003579. http://dx.doi.org/10.6019/PXD003579

**Value of the data**

• The data provides the so far largest proteomic profile of boar seminal plasma.
• The experimental approach used, SEC and 1-D SDS PAGE followed by NanoLC-ESI-MS/MS, is useful for identifying proteins in samples with a highly complex mixture of proteins such as seminal plasma.
• Comprehensive information and references of putative reproductive functionality of proteins identified in boar seminal plasma.
• This dataset can be used as a primary guide to characterize protein biomarkers for sperm quality and fertility in pig seminal plasma.

## 1 Data

A unique dataset is presented resulting from a qualitative and quantitative proteomic analysis of boar seminal plasma (SP), with more than 500 proteins listed and showing the relative abundance of a total of 432 proteins (Supplementary Table 1). Furthermore, an extensive description about their putative reproductive function is also provided, including appropriate references. This available information could help to a better understanding of the role of SP-proteins on boar sperm reproductive success.

## 2 Experimental design, materials and methods

In order to describe the boar SP-proteome, 33 entire ejaculates were collected, by using the semi-automatic collection device Collectis®, from 11 healthy and sexually mature Landrace and Large White boars (3 ejaculates per boar). Immediately after collection, ejaculated samples were centrifuged twice (1,500×*g* 10 min) to obtain SP sperm-free samples, which were stored at −80 °C until proteomic analysis. The 33 SP-samples were pooled and analyzed using a combination of SEC, 1-D SDS PAGE followed by NanoLC-ESI-MS/MS. The proteomics data and result-files from the analysis have been deposited to the ProteomeXchange Consortium [2] via the PRIDE partner repository, with the dataset identifier PRIDE: PXD003579 and doi:10.6019/PXD003579. Single SP-pools from each boar were analyzed by LC-SWATH-MS acquisition for determination of protein relative abundance.

### 2.1 Sample preparation

Seminal plasma samples were thawed at room temperature and ultracentrifuged (16,100×*g*, 4 °C, 1 min). Before starting the proteome analysis, the 33 SP-samples were split each one in two aliquots. One of them was mixed in a single pool for characterization the pig SP-proteome. Simultaneously, the second aliquots from each boar (*n*=3) were mixed, generating a total of 11 single pools (1 pool per boar).

### 2.2 Proteome analysis

The proteome analysis was performed as described in Ref. [1]. The more abundant proteins were identified from an aliquot of the mixed SP-sample analyzed by in-solution processing. The less abundant proteins were analyzed in-gel digestion processing using the portion of the 1-D SDS PAGE containing proteins with a molecular weight higher than 38 kDa obtained from the fractions collected after a SEC step. The digestion of the sliced gel was performed following the protocol used by Shevchenko et al. [3].

#### 2.2.1 LC-MS/MS analysis

The peptides recovered from in-solution and in-gel digestion processing were analysed as described in Ref. [1]. Briefly, peptides were examined by LC using a NanoLC Ultra 1-D plus Eksigent (Eksigent Technologies, Dublin, CA, USA) and the eluted peptides were direction-ionized using an ESI Nanospray III (AB SCIEX, Framingham, MA, USA) and then analyzed on an AB SCIEX TripleTOF 5600 mass spectrometer (AB SCIEX) in direct injection mode.

### 2.3 LC-SWATH-MS acquisition

For LC-SWATH-MS acquisition the TripleTOF 5600 (SCIEX) was configured as described by Gillet et al. [4] and later adapted to porcine SP by Perez-Patiño et al. [1]. Briefly, the mass spectrometer was operated in a looped product ion mode where the instrument was specifically tuned to allow a quadrupole resolution of Da/mass selection. The stability of the mass selection was maintained by the operation of the Radio Frequency (RF) and Direct Current (DC) voltages on the isolation quadrupole in an independent manner. A set of 37 overlapping windows, covering the mass range 450–1000 Da, was constructed using an isolation width of 16 Da (15 Da of optimal ion transmission efficiency and 1 Da for the window overlap). Consecutive swaths need to be acquired with some precursor isolation window overlap to ensure the transfer of the complete isotopic pattern of any given precursor ion in at least one isolation window and, thereby, to maintain optimal correlation between parent and fragment isotopes peaks at any LC time point. In this way, each single pool was loaded onto a trap column followed by an analytical column and eluted peptides were infused in the spectrometer nanoESI qQTOF (SCIEX TripleTOF 5600) operating in swath and in high sensitivity mode.

### 2.4 Data processing: protein identification, validation and quantification

After LC-MS/MS, The SCIEX.wiff data-files were processed using ProteinPilot v5.0 search engine (AB SCIEX). The Paragon algorithm (4.0.0.0, 4767) of ProteinPilot was used to search against the National Center for Biotechnology Information non-redundant (NCBInr; 70,353,186 proteins searched) protein sequence database with the following parameters: trypsin specificity, cys-alkylation (IAM), no taxonomy restricted, and the search effort set to through. To avoid using the same spectral evidence in more than one protein, the identified proteins were grouped based on MS/MS spectra by the Protein-Pilot Pro Group™ Algorithm, regardless of the peptide sequence assigned. The protein within each group that could explain more spectral data with confidence was depicted as the primary protein of the group. The resulting Protein-Pilot group file was loaded into PeakView® (v2.1, AB SCIEX) and peaks from SWATH runs were extracted with a peptide confidence threshold of 99% confidence (Unused Score ≥1.3) and a false discovery rate (FDR) less than 1%. The identified proteins were quantified using PeakView® from normalized label-free quantification (LFQ) intensity data.

## Gene ontology

Bioinformatic analysis of identified and validated SP-proteins was manually performed using the comprehensive bioinformatics tool for functional annotation UniProt KB database (www.uniprot.org) in combination with PANTHER (www.pantherdb.org). Both databases downloaded 06/04/2016, containing 63,686,057 and 1,424,953 entries in UniProt KB and PANTHER, respectively.

## Appendix A Supplementary material

Comprehensive dataset of the results achieved is provided in Supplementary Table 1. The dataset includes the SP-proteins identified and quantified highlighting the protein name, the specie, Unused Score, % coverage, accession number, number of peptides matched, UniProt ID, gene names, protein relative abundance (the highest and lowest values) and putative reproductive role.
